# Optimization of perioperative approaches for advanced and late stages of gastric cancer: clinical proposal based on literature evidence, personal experience, and ongoing trials and research

**DOI:** 10.1186/s12957-020-01819-6

**Published:** 2020-03-09

**Authors:** Maneesh Kumarsing Beeharry, Tian Qi Zhang, Wen Tao Liu, Zhu Zheng Gang

**Affiliations:** Department of Surgery, Shanghai Key Laboratory of Gastric Neoplasms, Shanghai Institute of Digestive Surgery, Rui Jin Hospital, Shanghai Jiao Tong University School of Medicine, Shanghai, 200025 China

**Keywords:** Advanced gastric cancer, Neoadjuvant therapy, Conversion therapy, Multimodality approach

## Abstract

**Background:**

The high incidence of gastric cancer (GC) and paradoxical high prevalence of advanced stage GC, amounting to around 2/3 at time of diagnosis, have urged doctors and researchers around the world not only to ameliorate the detection rate of GC at early stages but also to optimize the clinical management of GC at advanced stages.

**Content:**

We hereby recommend a more goal-oriented multimodality approach with objectives to increase survival rate and improve survival status. Based on precision and accurate clinical staging at diagnosis, we suggest that advanced stage GC (AGC) patients should be channeled into different treatment plans according to their disease status where they can be subjected to comprehensive measures involving chemo, radio, immunological, or target therapies depending on the pathophysiological behavior of their tumor. Patients assessed as potentially resectable cT4N + M0 can undergo neoadjuvant chemotherapy with intent of tumor downsizing and downgrading followed by surgery with intraoperative hyperthermic intraperitoneal chemotherapy (HIPEC) to decrease the incidence of peritoneal dissemination due to surgical trauma and adjuvant chemotherapy and radiation in cases of bulky nodal metastasis. In cases with distal metastasis, conversion therapy is recommended with the possibility of surgery of curative intent in case of favorable response. The options of alternate treatment options such as trans-catheter arterial chemoembolization (TACE) for limited liver lesions or neoadjuvant intraperitoneal plus systemic chemotherapy (NIPS) for peritoneal carcinomatosis have to be negotiated. With surgery as the cornerstone for cancer treatment, there is acknowledgment of the significance of perioperative comprehensive approaches but there has not been some consensus guiding clinical application. Henceforth, in this review, based on past literature, current guidelines and ongoing clinical trials, we have shared a proposal of the current treatment modalities in practice for the advanced stages of gastric cancer.

**Conclusion:**

Even though surgery is the golden standard of radical cancer treatment, clinical reality shows that without proper perioperative management, patients undergoing radical resections manifest high rates of recurrence and metastasis. Hence, in this review, we have outlined a clinical agenda to optimize the management of advanced stage GC with objective to improve survival outcome and quality of life of patients.

## Background

In spite of the epidemiological evidences suggesting a reduction in its incidence over the recent years, gastric cancer (GC) is yet the fifth most common malignancy and the third leading cause of cancer deaths in the world [1]. The high incidence of GC and paradoxical high prevalence of advanced stage GC (AGC), amounting to around 2/3 at time of diagnosis, have prompted doctors and researchers around the world not only to ameliorate the detection rate of GC at early stages but also to optimize the clinical management of GC at advanced stages [2]. The standard treatment option for the early and locally advanced stages of GC remains the complete resection of the lesion with acceptable margins with lymph node dissection [3]. However, surgery cannot be considered a standard procedure for patients with GC with metastasis unless the patients present with bleeding, obstruction, and perforation caused by the tumor, prompting for emergency palliative or salvage surgery [4]. When on one side, surgery can significantly improve the 5-year survival rate of early GC (EGC) to above 95%; on the other hand, surgery alone does not provide survival benefit in the locally advanced and late stages of GC with an approximate 5-year survival rate of 20 to 30% [5]. In quest to improve the prognosis for GC, more focus has been laid on the efficacy of comprehensive combined approaches, and hence, the concepts of postoperative adjuvant therapy, then neoadjuvant therapy and more recently, conversion therapy were developed [6–8]. Eventually, with the developments in precision medicine, the concept and role of immunotherapy in the adjuvant or neoadjuvant settings have been in the limelight of GC research [9]. With clear intent to improve the overall survival of GC patients, different treatment modalities have been developed and explored and in this paper, we have put forth our concept of optimization and standardization of perioperative therapy with respect to the recent updates and ongoing trials in the comprehensive approaches to the management of AGC.

Goal-directed perioperative therapy: comprehensive multimodality approaches to improve prognosis

### The postoperative adjuvant setting

With AGC described as T2-T4b/N0-3b/M0-M1 GC according to the 8th edition of AJCC-TNM classification [10–12], clinical evidence shows that its overall disease progression and recurrence rates after seemingly radical resection are around 25-50%, resulting in poor survival outcome [12]. In efforts to decrease the rate of metastasis after surgery of curative intent, the ACTS-GC and CLASSIC trials investigated and recommended that the addition of postoperative chemotherapy improved survival of AGC [13, 14]. Nevertheless, in spite of the efficacy of the 1-year treatment with S-1 or combination therapy with capecitabine and oxaliplatin for 6 months, approximately 20 to 30% of patients still developed metastasis or recurrence [3, 8]. With patients presenting with local recurrence and lymphatic metastasis, the ARTIST trial investigated the role of the postoperative chemo and radiotherapy combination in GC patients after D2 gastrectomy following postoperative chemotherapy with capecitabine and cisplatin (XP) with and without radiotherapy; even though radiotherapy did not demonstrate significant survival benefit, it reduced the rate of local metastasis by 6% and the subgroup analysis suggested that lymph node bulk load and Lauren classification were independent factors for survival advantage with adjuvant radiotherapy [15].

*Recommendation:* Based on these findings, the relative indications for postoperative setting therapy have been recommended as AGC with heavy lymph node bulk load can benefit from adjuvant chemoradiotherapy while in patients without heavy lymph node bulk; the addition of adjuvant chemoradiotherapy is optional.

### The preoperative neoadjuvant setting

The combination of surgery and adjuvant chemotherapy improved the outcome of AGC patients [13, 14] but due to relatively significant proportion of patients being diagnosed at advanced stages as result of the asymptomatic nature of GC, the relatively significant tumor burden and possible occult micrometastases challenge the radicality of a direct surgical approach [16]. With the potential benefits of primary tumor downstaging and lymph node metastasis and occult micrometastases control in GC patients with better tolerance in the preoperative stages, the concept of neoadjuvant chemotherapy (NAC) promised better understanding and control on the biological behavior of tumor progression and therapeutic response [17]. The Intergroup 0116 study was the first to show the significant overall survival benefits of adjuvant chemo radiation therapy for GC [18], and the next study was the MAGIC trial which evaluated the efficacy of perioperative adjuvant chemotherapy [19]. Although the findings from the Intergroup 0116 and the MAGIC trial were positive, following studies such as ARTIST and EORTC 40954 studies found no significant survival benefits for AGC, but EORTC 40954 demonstrated an increase in the radical resection rate in favor of T3-4N + M0 AGC undergoing NAC [15, 18]. In the FNCLCC/FFCD phase III trial, the 5-year survival rates were 24% in the surgery-alone arm and 38% in the perioperative chemotherapy arm (*p* = 0.02) [20]. In 2013, a Cochrane single patient data meta-analysis including 14 randomized trials showed an improvement in overall survival (HR = 0.81, 95% CI 0.79-0.89, *p* < 0.0001) with a 5-year survival gain of 9% with a 1.4 times radical resection rate favoring the NAC arm [21]. Recently, the German FLOT4 trial established the perioperative FLOT regimen increased rates of curative surgery and prolonged median PFS and median OS as compared with the ECF/ECX (epirubicin/cisplatin/oral capecitabine) regimen [22, 23]. Nevertheless, there are few ongoing clinical trials evaluating the implications of perioperative chemotherapy on the outcomes of AGC: The RESOLVE trial is a randomized, multicenter, controlled study to compare perioperative chemotherapy of oxaliplatin combined with TS-1 (SOX) versus SOX or oxaliplatin with capecitabine (XELOX) as postoperative chemotherapy (Identifier NCT01534546); The RESONANCE trial (Identifier NCT01583361) is comparing the efficacy of perioperative SOX versus surgery followed by adjuvant SOX, and the preliminary results showed that NAC improved the radical resection rate and did not increase the complication rate [24]. Another study, COMPASS-D compared two and four courses of NAC using S-1/CDDP (CS) or S-1/CDDP/docetaxel (DCS) followed by surgery and S-1 adjuvant chemotherapy and the preliminary results revealed that four courses of DCS improved the 3-year DFS [25]. FOCUS (IdentifierNCT01364376) is an ongoing trial comparing the efficiency of SOX versus FOLFOX in a neoadjuvant setting. RESOLVE 2 (Identifier NCT03691454) and MATCH (Identifier NCT02725424) are ongoing trial investigating the efficacy of SOX versus DOS with/without trastuzumab in a neoadjuvant setting [26].

Nevertheless, the indications for neoadjuvant chemotherapy have been varying in different countries, for e.g., In the USA, GC classified with >T2 infiltration and positive nodal metastasis are candidates for NAC. In China, the indications for NAC are locally advanced GC (T3 or T4 infiltration) with possible nodal metastasis while in Japan, the indications are GC cases assessed with high risk of recurrence (cIIIa to IIIc, Borrmann types III and IV) [27]. With goal to decrease and manage possible occult micrometastases in GC, the indications of perioperative therapy varies around the world since there are differences in the incidence of types of recurrence pattern between East Asian and Western patients with AGC: While locoregional recurrence and hematogenous metastasis are common in the USA, peritoneal, hematogenous, and lymph node metastasis are more common in East Asia [18, 28]. Therefore, adjuvant chemotherapy is the standard therapeutic strategy following resection in East Asia in contrast to standard adjuvant chemo radiation in the USA.

*Recommendation:* Based on current literature and clinical experience, we currently recommend NAC for patients clinically assessed as T3-4N + M0 with laparoscopic exploration to rule out the presence of peritoneal carcinomatosis at disease onset. We recommend that the choice of treatment regimen should be assessed by a designated panel of multidisciplinary team (MDT).

### Prophylactic hyperthermic intraperitoneal chemotherapy

Koga et al. first reported the use of hyperthermic intraperitoneal chemotherapy (HIPEC) as a prophylaxis against peritoneal dissemination due to surgical trauma or disease progression in 1988 where significant improvement was noted in the 3-year survival and peritoneal recurrence rates [29]. As from 1992 to 2002, 128 GC patients with peritoneal dissemination underwent surgery in our hospital were included in an HIPEC experiment and the 5-year survival rates were 5.5% for patients in the resection group and 0% for patients in the non-resection group, and HIPEC was an independent prognostic factor by multivariate analysis [30]. In another trial from our institution (1998 to 2001), it was found that when comparing 42 subjects who underwent surgery + HIPEC with 54 subjects who had surgery alone, the 1-, 2-, and 4-year survival rates were more favorable in the HIPEC group, and the peritoneal recurrence was 34.7% vs. 10.3%, in favor of HIPEC [31]. In a study by Cui et al., where 192 AGC patients were randomly divided into the following four groups (control, NAC, HIPEC and combined groups), the results suggested that NAC combined with HIPEC for the treatment of AGC is well tolerated and exhibits improved compliance and efficiency [32]. In another study from our team, with a pool of 80 patients with 40 patients undergoing prophylactic HIPEC while the control group had surgery alone, the results showed that the experimental arm had significant survival benefit with a decrease in peritoneal metastatic rate [33].

**Fig. 1:**
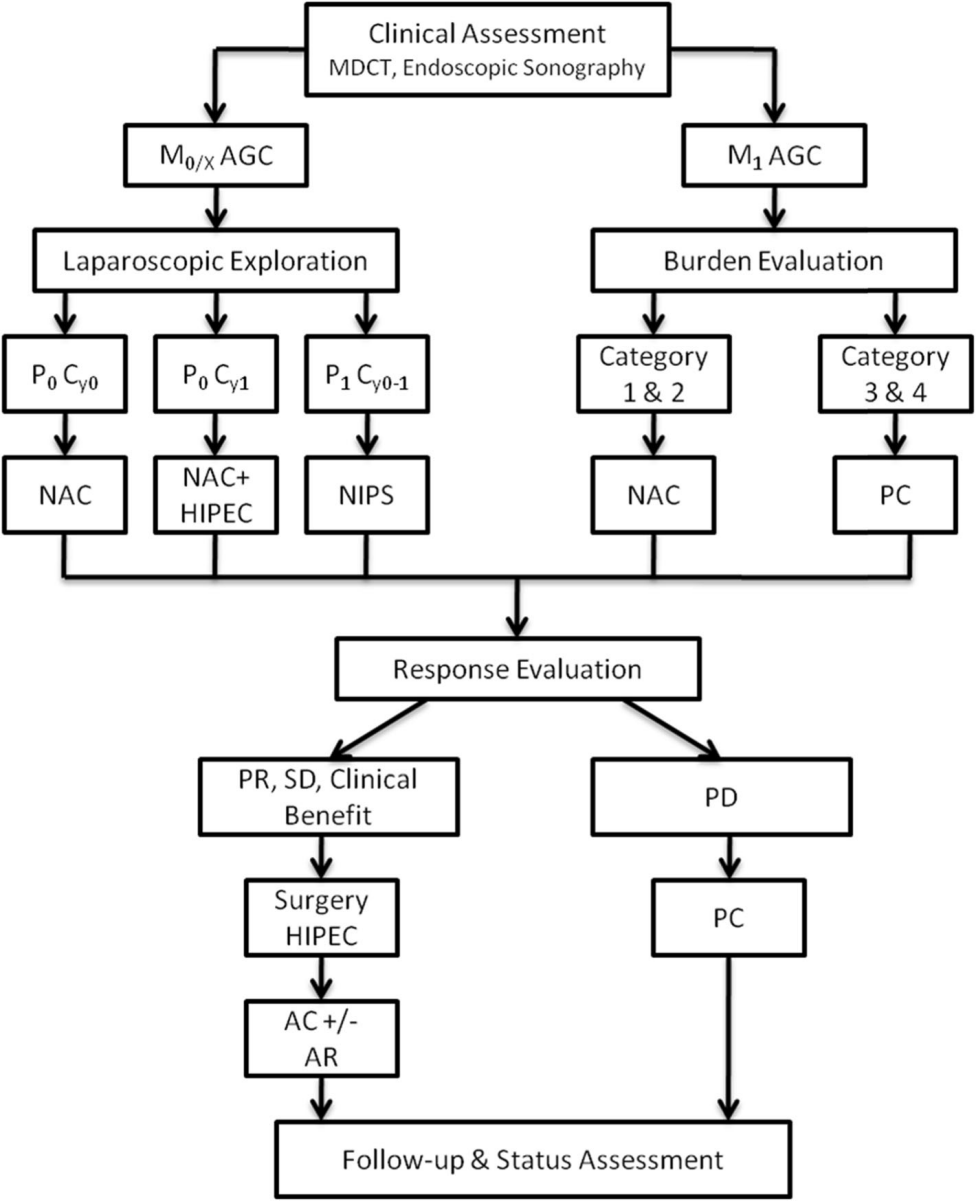
Optimized clinical management of AGC based on current evidence and personal experiences. M_0/X_ AGC refers to AGC without macroscopic metastasis; M_1_ AGC refers to AGC with clinical macroscopic metastasis; P_0_ C_y0_ refers to no macroscopic peritoneal metastasis and no positive cytology; P_0_ C_y1_ refers to no macroscopic peritoneal metastasis but positive cytology; P_1_ C_y0-1_ refers to macroscopic peritoneal metastasis with or without positive cytology; NAC refers to neoadjuvant combined therapy; HIPEC refers to hyperthermic intraperitoneal chemotherapy [34]; NIPS refers to neoadjuvant bidirectional intraperitoneal/systemic chemotherapy [35]; PC refers to palliative combined therapy; PR refers to partial remission, SD refers to stable disease, and PD refers to progressive disease according to RECIST evaluation criteria; AC refers to adjuvant combined therapy; AR refers to adjuvant radiotherapy

*Recommendation:* While the efficacy of perioperative chemotherapy has been largely investigated and recognized, the concept of HIPEC as a prophylaxis against PC in the clinical management of AGC is yet recent (Fig. 1). Henceforth, since AGC patients with serosal involvement are at risk of occult peritoneal dissemination due to surgical trauma or transplantation of free cancer cells (FCC), we recommend intraoperative HIPEC at time of surgery of curative intent. With the rationale of regional chemotherapy, mechanical erosion, and FCC down-gradient and hyperthermia, there are strong evidences of the role of HIPEC in the management of AGC.

### Conversion therapy for non-resectable late stage GC

The common indications for palliative surgery remain perforation, bleeding, or obstruction due to the tumor itself. Otherwise, the role of surgery in AGC is not regarded as radical. However, with the developments and improvements in antitumor treatment such as chemotherapy, radiotherapy, or immunotherapy, the concept of conversion or salvage surgery came into the limelight in cases that could transition from non-resectable to resectable GC with an aim to improve survival and quality of life of the patient [36, 37]. In the aftermath of the REGATTA trial, even though some authors emphasized the beneficial role of palliative gastrectomy, Fujitani et al. described no survival benefit was noted for palliative gastrectomy prior to chemotherapy [38]. Given the poor results achieved with chemotherapy alone, drug resistance or cumulative adverse effects, the possible benefits of surgical resection as a part of a multimodality treatment strategy for selected patients who responded well to the chemotherapy were evaluated [39–41]. In a study by Yoshida et al., conversion therapy was classified into four categories defined with respect to the biological and heterogeneous characteristics of GC [42, 43]: category 1—in cases where the primary tumor and the metastatic lesion are evaluated technically resectable with good oncological margins, primary tumor resection with metastasectomy is recommended with or without NAC [42–44]; category 2—in cases where metastatic lesions are considered to be oncologically or technically unresectable, such as multiple liver metastases, liver lesions infiltrating the hepatic and/or portal vein or distant LN metastasis, first-line chemotherapy is recommended as the induction chemotherapy and in case of good therapeutic response with tumor regression; conversion surgery can be considered [44]; category 3—in cases of bulky masses or bulky metastatic lesions, palliative combined therapy is recommended and surgical intervention is to be considered only in circumstances of local palliation needs [45]; and category 4—in GC with extensive metastasis without signs of significant therapeutic benefit, palliative combined therapy is the best recommendation. Nevertheless, as far as the unresectable GC with peritoneal carcinomatosis, the PHOENIX trial revolutionized clinical practice with the introduction of the neoadjuvant intraperitoneal and systemic chemotherapy (NIPS) treatment regimen where after the combination of oral + intravenous + intraperitoneal chemotherapy followed by conversion surgery, the 1-year survival could be increased to 56.0% to 80.0% [46–49].

*Recommendation:* Based on updated literature, potentially resectable late stage GC cases have the indications of conversion therapy: Our team recommends that after precise analysis by a multidisciplinary team, the patient should be subjected to first-line chemotherapy with or without radiotherapy. If the patient presents with peritoneal carcinomatosis, the NIPS treatment regimen is recommended. In cases with clear liver lesions only, chemotherapy with trans-arterial chemoembolization (TACE), is recommended. For all these cases, regular follow-up and radiological evaluations are recommended. In cases of disease partial or complete remission, salvage surgery is recommended.

### Personal experiences and ongoing studies

The concept of “non-resectable” GC needs not only to be assessed from the surgical technique point-of-view but also from the perspective of oncology. When the goal of treatment is better survival, the course of treatment needs to be evaluated and assessed by the response rate (RR) and performance status of the patient. In a study of our faculty, a study involving 37 cases of advanced GC patients using the combination of docetaxel, oxaliplatin, and capecitabine as first-line treatment revealed a RR of 29.7% and a disease control rate of 91.9% [50–52]. Furthermore, by accentuating preoperative therapy and meticulously observing the changes in the physio-biological behavior of the tumor during the disease course and then favoring surgery can significantly improve the outcome of such initially “non-resectable” advanced GC. Based on the Japanese study PHOENIX, our institution initiated a phase II clinical trial to investigate the efficacy and safety of the NIPS regimen and our findings suggested a conversion gastrectomy rate of 72.7% with a 1-year OS rate was 63.6%, where the 1-year OS rate of the patients with conversion gastrectomy and the patients with stage P3 reached to 87.5% (7/8) and 50.0% (4/8), respectively [53]. Following these results, we are currently recruiting patients to the phase III multicentre Dragon clinical trial (Chinese Clinical Trial Registration ID ChiCTR-IIR-16009802) for patients presenting with PC at time of diagnosis. On one side where the Dragon I trial is assessing the treatment efficacy of the multimodality of NIPS in GC patients with PC, another trial involving the application of neoadjuvant laparoscopic HIPEC to treat occult PC combined with NAC for tumor downstaging followed by surgery with intraoperative HIPEC to prevent peritoneal dissemination due to surgical trauma as a prophylaxis comprehensive approach for AGC at high risk of PC has been initiated (Chinese Clinical Trial Registration ID ChiCTR1900024552).

## Optimization of the comprehensive management of AGC based on personal experiences

According to guidelines, EGC patients would undergo radical resection. On the other hand, AGC patients should be diverted into two different treatment streams (neoadjuvant and conversion) depending on their clinical assessments. Patients without imageologically proven metastases should be subjected to standard laparoscopic exploration to rule out peritoneal insemination. In case of no peritoneal involvement, the patients should be recommended for neoadjuvant combined therapy, patients with no macroscopic metastases but positive cytology could undergo NAC involving HIPEC, with as shown in a meta-analysis by Coccolini et al., can significantly improve the 1-, 2-, and 3-year survival after with positive effects on peritoneal recurrence [54]. Patients with macroscopic metastases with or without positive cytology would be suitable candidates for the Dragon I clinical trial involving the NIPS regimen treatment. On the other hand, patients with macroscopic metastases should be categorized according to the stratification suggested by Yoshida et al. [43]. Categories 1 and 2 patients would be subjected to neoadjuvant combined therapy with goal to create the conditions for R0 resection while categories 3 and 4 would be subjected to palliative therapy. After definite cycles of therapy, in case the patients from either group showed signs of favorable response, including partial remission (PR), stable disease (SD) with clinical benefit, the patient could be recommended to radical surgery followed by comprehensive adjuvant combined therapy. On the other hand, if the patient showed signs of disease progression (PD), then palliative therapy should be recommended.

### Conversion therapy with molecular-targeted therapy and immunotherapy

When the tumor shows human epidermal growth factor receptor 2 (HER2) positivity, regimens combined with trastuzumab is promising as a neoadjuvant chemotherapy. The JCOG1301 phase III study is currently underway to evaluate the efficacy of neoadjuvant therapy using trastuzumab in combination with S-1/cisplatin for the bulky N population [55, 56]. A fully human IgG4 programmed death 1 (PD-1) immune checkpoint inhibitor antibody has demonstrated survival benefits for various tumors. ATTRACTION-05 phase III study is ongoing to evaluate the efficacy of nivolumab in combination with postoperative chemotherapy (S-1 or capecitabine plus oxaliplatin) in AGC [57]. KEYNOTE-585 phase III study is also underway to evaluate the efficacy of pembrolizumab in combination with perioperative chemotherapy (cisplatin with either oral capecitabine or intravenous 5-fluorouracil) for patients with AGC and esophagogastric junction cancer [34]. Although systemic chemotherapy is still the mainstay treatment of metastatic disease, the introduction of agents targeting HER2 and vascular endothelial growth factor (VEGF) has brought this disease into the molecular and personalized medicine era. The preliminary yet encouraging clinical efficacy observed with immune checkpoint inhibitors, e.g., anti-PD-1/PD-1, will further shape the treatment landscape for GC [56].

Recent significant advances in understanding the gastric cancer disease process from both biological and genomic prospective have brought target-oriented therapy for advanced gastric cancer into clinical research and practice. The cancer genome atlas (TCGA) project performed comprehensive molecular characterization of gastric adenocarcinoma and identified four major molecular subtypes as Epstein–Barr virus (EBV)-infected tumors, microsatellite instability (MSI) tumors, genomically stable tumors, and chromosomally unstable tumors [35]. Further genomic alterations and molecular subtypes/sub-classifications may help guide the therapeutic implications for gastric cancer [9, 58–60].

## Conclusion

The high incidence and yet relatively poor prognosis of gastric cancer have urged researchers around the world to investigate measures guiding to a more comprehensive clinical management of the disease especially at advanced and late stages. Based on available evidence, ongoing research and trials and personal experience, we recommend a more goal-oriented multimodality approach with objectives to increase survival rate and improve survival status. Based on precision and accurate clinical staging at diagnosis, we suggest that AGC patients should be channeled into different treatment plans according to their disease status where they can be subjected to comprehensive measures involving chemo, radio, immunological, or target therapies depending on the nature of their tumor. We believe that such standardization would optimize the perioperative management for AGC patients, with a goal to improve prognosis and survival.

## Data Availability

Data and materials are available on demand.
